# Intrauterine device migration into the bladder with urolithiasis: A case report

**DOI:** 10.1016/j.ijscr.2025.111409

**Published:** 2025-05-10

**Authors:** Yassine Jaziri, Bilel Saidani, Ahmed Saadi, Ayed Haroun, Marouene Chakroun, Riadh Ben Slama

**Affiliations:** The Faculty of Medicine of Tunis, Tunis, Tunisia; The Department of Urology, Charles Nicole Hospital, Tunis, Tunisia

**Keywords:** Intrauterine device migration, Cystolithiasis, Bladder stones, Case report

## Abstract

**Introduction:**

Intrauterine devices (IUDs) are the contraceptive method of choice for many women across the globe. Extrauterine migration of IUDs is a rare complication, with the intravesical location and vesical stone formation being an exceptional event.

**Case presentation:**

We report a case of a 39-year-old woman with a history of IUD insertion four years prior to the onset of lower urinary tract symptoms consisting mainly of dysuria and pollakiuria. A migrated device was discovered in the bladder, resulting in stone formation. The bladder stone was successfully treated using laser lithotripsy with an uncomplicated recovery and the resolution of all symptoms at further check-up.

**Discussion:**

IUD intravesical migration with stone formation around the device is a rare but noteworthy complication of the contraceptive method, especially in the context of recurring urinary tract symptoms. Ultrasonography and standard radiography can be helpful tools in diagnosing device migration. Treatment options can differ, with laser lithotripsy being a minimally invasive and efficient method to treat the resulting calculus around the migrated device and for device extraction. Post-operative follow-up should focus on symptom resolution as well as the presence of fistulae.

**Conclusion:**

While IUDs remain an efficient and accessible method of contraception, complications including device migration into the bladder with or without stone formation should be considered, particularly in the context of recurring lower urinary tract symptoms, and should be treated immediately to avoid further complications.

## Highlights

• A rare case of a migrated intrauterine device with stone formation was diagnosed.
• Lower urinary tract symptoms were the main clinical manifestation of this rare complication.
• Radiography and ultrasonography were efficiently used to make the diagnosis of the migrated device.
• The bladder stone was fragmented using laser lithotripsy and the device removed endoscopically.
• The case highlights the importance of post-insertion follow-up of DIUs to detect complications.

## 1 Introduction

An intrauterine device is a common and easy contraceptive method that has proved to be effective in preventing unwanted pregnancies [1]. IUD migration outside the uterus is a rare complication, with intravesical migration being rarely spoken of in literature [2].

We report the case of an intrauterine device migration into the bladder with cystolithiasis, with the diagnosis made based on medical history, clinical examination, imaging and endoscopic findings. We describe the diagnosis and treatment processes.

## 2 Case presentation

This was the case of a 39-year-old Tunisian woman, who consulted our department in November 2024 for recurring lower urinary tract symptoms consisting of dysuria and pollakiuria evolving over a period of six months, with no reported hematuria. The patient presented no relevant medical or family history of lithogenesis. She had a regular menstrual cycle of 28 days, no prior history of intrauterine procedures and had three pregnancies, three natural births and no abortions, with the last birth taking place at the age of 32. The patient had a copper-T intrauterine device inserted by her gynecologist, four years prior to the onset of symptoms, with no further follow-up. The clinical examination showed only a slight hypogastric tenderness. A gynecological exam showed absence of the IUD strings in the vagina. A transvaginal ultrasound showed absence of the device in the uterine cavity.

Urinalysis results showed a low white blood cell count, and there was no bacterial growth in urine culture. Blood tests showed a normal white blood cell count and negative inflammation markers, as well as a normal renal function.

**Fig. 1:**
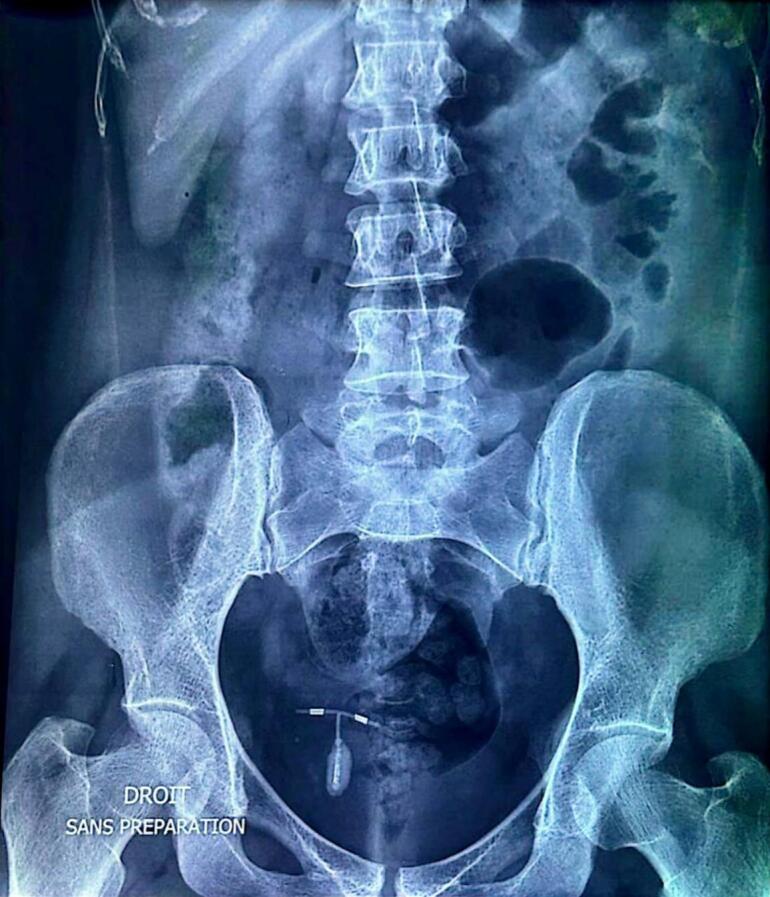
Abdomen X-ray showing intrauterine device calcification.

An abdominal ultrasound and an abdomen X-ray (Fig. 1) were performed, showing the migrated intrauterine device inside the bladder with a large calcification of 22 ∗ 12 mm covering most of the surface of its vertical arm.

**Fig. 2:**
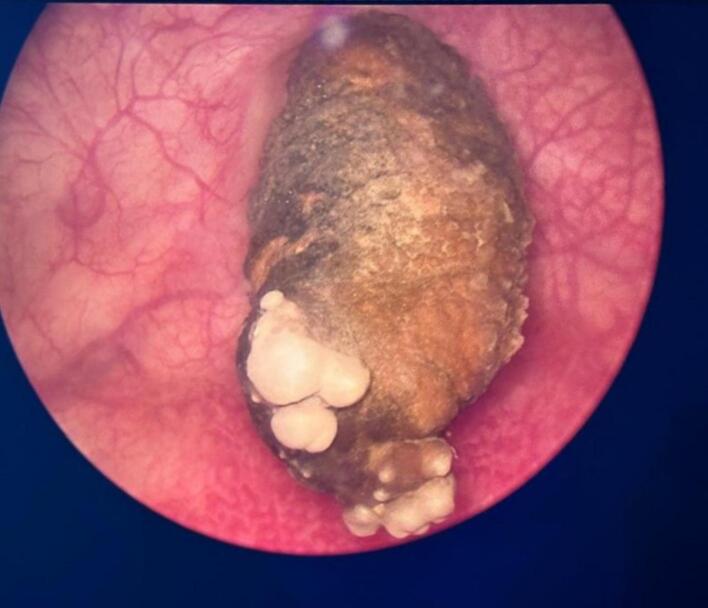
Cystoscopic view of the bladder stone before fragmentation.

**Fig. 3:**
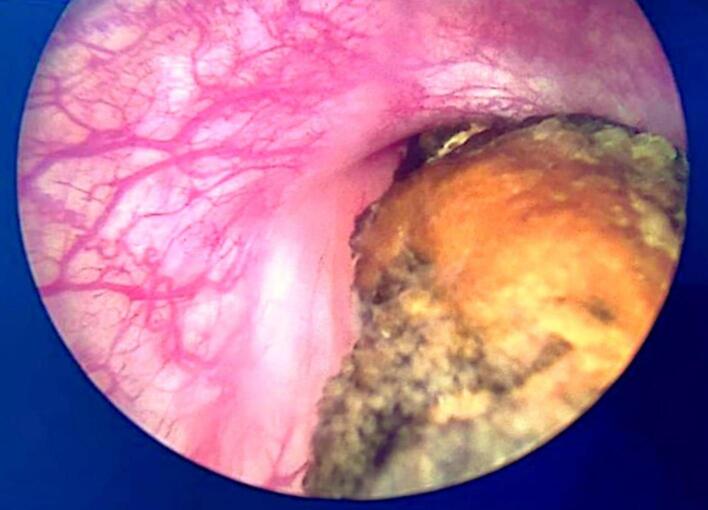
IUD insertion into bladder wall with a segment encrusted through a 1 cm orifice.

**Fig. 4:**
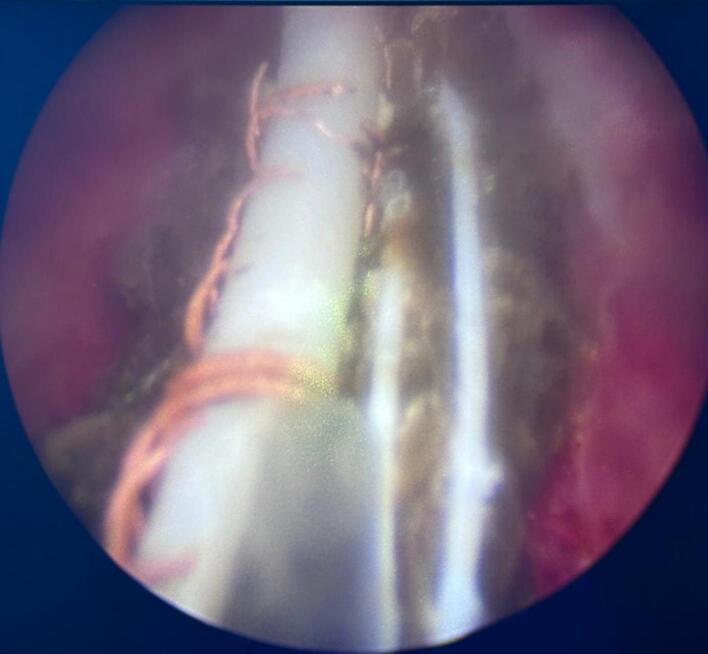
Exposing intrauterine device after laser lithotripsy.

**Fig. 5:**
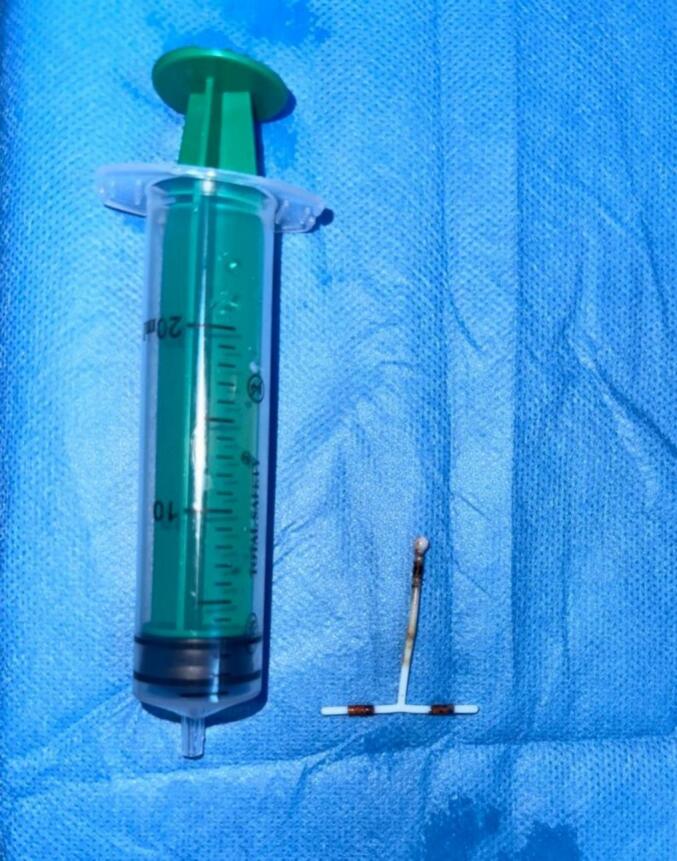
IUD specimen following cystoscopic extraction from the bladder.

We decided to admit the patient to our department. An initial cystoscopy was performed that showed a large stone that was adherent to the bladder wall (Fig. 2). It seemed that one part of the device was encrusted in the posterior vesical wall through an orifice of 1 cm of diameter (Fig. 3). We performed a YAG Holmium Laser lithotripsy of the calcified part of the IUD and exposed the device in its entirety (Fig. 4). The device was then extracted using endoscopic forceps in its totality, with no incident (Fig. 5). No apparent fistula was present after extraction. However, given the presence of the orifice, a transurethral catheter 20 Fr was then inserted to be left in for ten days. The patient was later discharged within 24 h. At follow-up a month later, the patient reported a complete resolution of all symptoms. No urinary or vaginal leakage was reported.

## 3 Discussion

Intrauterine devices are an effective contraceptive method that offers a number of advantages, mainly their easy access, low cost, long-term use and low risk of complications [1]. The two types of IUDs mainly used are the hormonal or levonorgestrel-releasing IUD and the copper-based Cu380A IUD, both of which cause a local inflammatory response that makes the uterine cavity inhospitable to fertilization and implantation, hence the contraceptive effect [1]. However, it is that same inflammatory mechanism that can allow for the migration of the device outside the uterine cavity, with the device making its way gradually through the different layers of the uterus and the bladder [2].

IUDs carry the risk of infection, perforation, expulsion and ectopic pregnancy [1]. Perforation risk is maximal at the time of insertion, but can also occur at a later time, hence the importance of a proper insertion technique and post-insertion follow-up [3]. For our patient, the device used was the copper-T device with an insertion duration of four years without any follow-up.

Device migration or translocation is a rare but noteworthy complication that can have severe consequences, with an incidence ranging from 0.2 to 9.6 per 1000 insertions, although most cases remain asymptomatic. Many migratory locations have been described in literature, including, but not limited to the peritoneum, the omentum and the bowels [4,5]. Migration into the bladder is a rare case, estimated at less than 2 % of all IUD perforations, but remains, nonetheless, a serious event that can have severe complications [4,5]. The migratory process is generally a slow one, spanning over the course of many years, in contrast to only four years of IUD insertion in the case of our patient, well within the accepted device duration of ten years [6]. Bjørnerem even described a case where the IUD complete intravesical translocation happened within a mere three weeks [5]. Once the device is inside the urinary bladder and in direct contact with urine, it can favor lithogenesis.

Intravesical migration of the IUD can be completely asymptomatic, which would explain its late detection, or it can manifest by irritative lower urinary tract symptoms, abdominal pain, hematuria or recurrent urinary infections [2]. In the case of our patient, dysuria, pollakiuria and abdominal discomfort were reported. IUD migration into the bladder therefore needs to be suspected in patients using the contraceptive method, who are experiencing recurrent lower urinary tract symptoms.

In our case, standard X-ray imaging and ultrasound proved efficient in diagnosing the migration of the intrauterine device with stone formation. A cystoscopy should always be performed, as it offers a direct visualization of the device and allows for therapeutic procedures such as laser lithotripsy when indicated.

Management of IUD migration inside the bladder with stone formation necessarily requires the treatment of the bladder stone, and eventually the removal of the device to prevent recurrence and allow the treatment of any resulting fistula or lesions. The World Health Organization recommends the removal of any migrated intrauterine device immediately after its detection, regardless of type or location [4].

Numerous treatment plans have been described in this regard, ranging from open surgery with stone and device extraction and cystorrhaphy, to minimally invasive laparoscopic and endoscopic approaches [[7], [8], [9]]. We chose the endoscopic approach, given the low morbidity and high efficiency associated with the technique, as well as our team's experience in these procedures [10]. In our case, our approach consisted of an initial cystoscopy associated with laser lithotripsy of the bladder stone that had formed around the device, which allowed its extraction using endoscopic forceps with minimal lesions to the bladder wall. We recommend using this minimally invasive procedure as a first-line treatment in such scenarios. It allowed the patient to make a full recovery and a complete disappearance of the aforementioned symptoms.

Postoperative follow-up should particularly pay attention to the resolution of symptoms and to the presence of any persistent fistulae through clinical control for leakage. Schwartzwald reported a case of a vesicouterine fistula with menouria (vesical menstruation) that did not resolve until the fistula was repaired and the uterus removed [11], while Wallace and Wilson reported a rare but noteworthy case of an enterovesical fistula caused by a migrated IUD that failed to resolve by conservative urethral catheter management and required robotic fistula excision and small bowel resection [12].

## 4 Conclusion

Intrauterine device migration into the bladder can be a serious complication and should be suspected in patients with an IUD, who are experiencing recurring lower urinary tract symptoms. Standard radiography and ultrasonography can be helpful tools in detecting translocated intravesical IUDs. Minimally invasive endoscopic treatment with laser fragmentation can be an effective therapeutic approach in treating migrated intrauterine devices with calcification. Postoperative follow-up should pay attention to the presence of fistula.

## SCARE guidelines

This work has been reported in line with the SCARE criteria [13].

## Author contribution

*Mr. Yassine Jaziri: corresponding author*

Conception and design of the paper

Data collection

Data analysis and interpretation,

Drafting of the manuscript

Revision of the manuscript

*Mr. Bilel Saidani:*

Conception and design of the paper

Data collection

Data analysis and interpretation,

Revision of the manuscript

*Mr. Ahmed Saadi*

Data collection

Data analysis and interpretation,

Revision of the manuscript

*Mr. Haroun Ayed*

Data collection

Data analysis and interpretation,

Revision of the manuscript

*Mr. Marouene Chakroun*

Data collection

Data analysis and interpretation,

Revision of the manuscript

## Consent

Written informed consent was obtained from the patient for the publication of this case report and any accompanying images. A copy of the written consent form is available for review by the Editor-in-Chief of this journal upon request.

## Ethical approval

This work is exempt from ethical approval as per the policies of the concerned hospital.

## Guarantor

Yassine Jaziri

## Research registration number

N/A

## Funding

No sources of funding were used in the course of this work.

## Conflict of interest statement

The authors declare that no competing financial interests or personal relationships have influenced the work reported in this paper.
